# A scoping review of cognitive assessment tools and domains for chemotherapy-induced cognitive impairments in cancer survivors

**DOI:** 10.3389/fnhum.2023.1063674

**Published:** 2023-02-20

**Authors:** Kazuya Saita, Satoru Amano, Fumiko Kaneko, Hitoshi Okamura

**Affiliations:** ^1^Department of Psychosocial Rehabilitation, Graduate School of Biomedical and Health Sciences, Hiroshima University, Hiroshima, Japan; ^2^Department of Rehabilitation, School of Allied Health Sciences, Kitasato University, Sagamihara, Kanagawa, Japan

**Keywords:** cognitive disorder, cognitive function, cancer-related cognitive impairment (CRCI), ICF, neoplasm, chemo brain, CICIs

## Abstract

**Backgrounds:**

Cancer survivors suffer from specific symptoms known as chemotherapy-induced cognitive impairments (CICIs). CICIs are difficult to capture with existing assessments such as the brief screening test for dementia. Although recommended neuropsychological tests (NPTs) exist, international consensus and shared cognitive domains of assessment tools are unknown. The aim of this scoping review was as follows: (1) to identify studies that assess CICIs in cancer survivors; (2) to identify shared cognitive assessment tools and domains by mapping the domains reported in studies using the International Classification of Functioning, Disability and Health (ICF) framework.

**Methods:**

The study followed the recommendations made by the Preferred Reporting Items for Systematic Reviews and Meta-Analyses extension for Scoping Reviews. We searched the following three databases through October 2021: PubMed, CINAHL, and Web of Science. Prospective longitudinal or cross-sectional studies were selected to determine CICI-specific assessment tools for adult cancer survivors.

**Results:**

Sixty-four prospective studies (36 longitudinal studies and 28 cross-sectional studies) were included after checking for eligibility. The NPTs were divided into seven main cognitive domains. The specific mental functions were often used in the order of memory, attention, higher-level cognitive functions, and psychomotor functions. Perceptual functions were used less frequently. In some ICF domains, shared NPTs were not clearly identified. In some different domains, the same NPTs were used, such as the trail making test and the verbal fluency test. When the association between the publishing year and the amount of NPT use was examined, it was found that the amount of tool use tended to decline over the publication years. The Functional Assessment of Cancer Therapy-Cognitive function (FACT-Cog) was a shared consensus tool among the patient-reported outcomes (PROs).

**Conclusion:**

Chemotherapy-induced cognitive impairments are currently gaining interest. Shared ICF domains such as memory and attention were identified for NPTs. There was a gap between the publicly recommended tools and the tools actually used in the studies. For PROs, a clearly shared tool, FACT-Cog, was identified. Mapping the domains reported in studies using the ICF can help in the process of reviewing consensus on which NPTs may be used to target cognitive domains.

**Systematic review registration:**

https://center6.umin.ac.jp/cgi-open-bin/ctr/ctr_view.cgi?recptno=R000053710, identifier UMIN000047104.

## 1. Introduction

There has been a continuous increase in cancer patients worldwide, with 19.3 million new cancer patients, and almost 10 million cancer deaths, as estimated in 2020 (Sung et al., 2021). With the early detection of cancer and state-of-the-art treatments, the number of cancer survivors has tended to increase. Common impairments in cancer survivors are general physical impairments (such as somatic pain, fatigue, weakness, and deconditioning), specific physical impairments (such as lymphedema, shoulder pain, and sensory deficits), and psychosocial functional impairments (Silver et al., 2013). With many cancer survivors expected to return to work, or engage with society, the presence of cognitive impairments can largely inhibit their ability to do so.

“Cancer-related cognitive impairment (CRCI)” has been used as a general term for impairments in patients with cancer. This includes the many factors influencing cognitive function including cancer, cancer treatments (surgery, radiotherapy, chemotherapy, targeted therapy, hormonal therapy, and immunotherapy), psychological factors (depression, anxiety, and fatigue), genetic polymorphisms, and psychosocial factors (education level, cognitive reserve, and age) (Lange et al., 2019). Understanding the phenomenon of CRCI is getting more difficult due to the confounding factors. CICIs are the most frequently encountered and unresolved treatment-related consequence. Several review studies have investigated not only the effects of chemotherapy, but also the cognitive dysfunction associated with concomitant radiation and hormone therapy (Lindner et al., 2014; Bernstein et al., 2017; Bray et al., 2018). Focusing on CICIs may lead to a stereotypical understanding of the phenomenon of CRCI.

Previous studies have reported that cancer survivors experience cognitive decline in the range of 16–75%, before and after chemotherapy (Wieneke and Dienst, 1995; Wefel et al., 2004; Fan et al., 2005; Janelsins et al., 2017). This discrepancy in the range of estimates of cognitive impairments may be due to lack of widely shared cognitive assessment tools (Whitford et al., 2020). Neuropsychological tests (NPTs) and patient-reported outcomes (PROs) are frequently used to assess the CICIs. NPTs provide an objective evaluation of generalized or specific cognitive function in brain-damaged patients. Three NPTs have been recommended for detecting the CRCI by the International Cognition and Cancer Task Force (ICCTF): trail making test (TMT), Hopkins Verbal Learning Test-revised (HVLT-R), and the controlled oral word test (COWA) (Wefel et al., 2011). In contrast, subjective evaluations, such as cognitive complaints, are acknowledged as crucial evaluations. This is because there have been reports of difficulties in real-life situations even though the traditional NPTs have not detected clear cognitive impairments. PROs are a subjective assessment tool that can most sensitively capture cognitive changes and complaints. On the downside, PROs are sensitive to psychosocial effects such as anxiety and depression (Janelsins et al., 2017). Due to cultural and regional diversity, the NPTs and PROs in use are expected to have a wide range of variations. In this respect, it is necessary to investigate what assessment tools are being used as international consensus tools. Additionally, the ICCTF recommends that CRCI studies be investigated as follows: (1) longitudinal studies, (2) compared to healthy controls, and (3) correlated with neuroimaging (Deprez et al., 2018). In particular, neuroimaging assessment can reveal structural changes in diffusion tensor imaging associated with chemotherapy (Deprez et al., 2013). Therefore, we included the studies that employed neuroimaging evaluations in this review.

Although various classification methods have already been proposed for cognitive domains, the use of the International Classification of Functioning, Disability and Health (ICF) framework is a valuable attempt. The ICF framework is the World Health Organization (WHO) classification scheme used internationally to describe the functional status about health conditions (Cieza et al., 2002). The framework consists of the following components: Body functions (b) and Structures (s), Activities and Participation (d), Environmental factors (e), and personal factors. These components are coded using letters and numbers and are widely applied in various research fields including health, medical, welfare, and education (Cieza et al., 2002) (e.g., attention functions, b140). We would be able to share our insights with healthcare professionals from various fields if we could categorize cognitive assessment tools using the cognitive domains of the ICF framework.

The aims of this review were (1) to identify studies that evaluate CICIs; (2) to identify cognitive-related assessment tools used in these studies and the domains they target; and (3) to identify common tools by mapping the cognitive domains reported in the studies using a common ICF framework.

## 2. Materials and methods

This review followed the Preferred Reporting Items for Systematic Reviews and Meta-Analyses extension for Scoping Reviews (PRISMA-ScR) recommendations (the completed checklists were provided in Supplementary material A; Tricco et al., 2018). Our protocol was registered with the University Medical Information Network (UMIN) Center (UMIN000047104).

### 2.1. Eligibility criteria/information sources

We searched three online databases: PubMed, CINAHL, and Web of Science. Search limits included peer-reviewed studies, whether they were published in English, and were published by October 1, 2021. The inclusion criterion for the present review was that the studies must have principally investigated the effects of chemotherapy on cognition in adults with cancer. However, studies that investigated other cancer treatments separately from chemotherapy were included if they had a distinct chemotherapy treatment group. The exclusion criteria for our study were as follows: (1) no abstract, (2) languages other than English, (3) review article, interventional trial, and case study, (4) experiments with animals, (5) primary brain tumor or brain metastasis, (6) unclear distinction between hormonal therapy and radiotherapy, (7) having any other reason that the reviewer finds to be an obvious content discrepancy. An example of this content inconsistency is when there is a claim to an exclusive examination of the effect of chemotherapy treatment on cognitive impairment, but reviewers have considered the impairment to be strongly influenced by depression, anxiety, fatigue, or anemia.

### 2.2. Search strategy/data charting

We used the Medical Subject Heading (MeSH) and keyword terms as the targeted search strategy for literature. Subject headings and synonyms were used to expand the search and were combined using boolean operators (i.e., AND, OR) and truncations (i.e., cognit*). The MeSH terms used to search the literature using PubMed and CINAHL online databases were as follows: “cognition disorders”[MeSH Terms] OR (“cognition”[All Fields] AND “disorders”[All Fields]) OR “cognition disorders”[All Fields], AND “neoplasm’s”[All Fields] OR “neoplasms”[MeSH Terms] OR “neoplasms”[All Fields] OR “neoplasm”[All Fields], AND “drug therapy”[Subheading] OR (“drug”[All Fields] AND “therapy”[All Fields]) OR “drug therapy”[All Fields] OR “drug therapy”[MeSH Terms] OR (“drug”[All Fields] AND “therapy”[All Fields]). The keywords hierarchized by MeSH terms are shown in Supplementary material B. Other search terms in the online databases were as follows: “cancer” AND “cognit*” AND “adjuvant chemotherapy” AND “related” OR “induced.”

Duplicate articles were excluded from all papers obtained by this search method. The articles were then independently reviewed by two reviewers (KS and SA) to check for the inclusion/exclusion criteria by title, abstract, and keywords. After downloading or ordering the full text of the report, the data were again thoroughly examined and extracted by the two reviewers. In cases where the necessary information for charting was not available, we contacted the authors concerned to identify additional sources. Disagreements between the two reviewers at each step were either resolved by consensus after reviewing the full text or by a procedure in which a third senior researcher (HO) made the final decision.

### 2.3. Data items/synthesis of results

All data were extracted in equal parts from the reports of two of the review authors (KS and SA). The following information was extracted from each article: first author, year of publication, country of the first author, type of study, sample size at baseline, type of cancer, and full description of the cognitive assessment performed (cognitive assessment tools and instruments used, cognitive domains considered by the authors). The sample size was shown after excluding healthy participants who did not receive chemotherapy. The cognitive assessment tools were organized by the components of standard NPTs as objective assessments, and PROs as subjective assessments. The type of neuroimaging devices used was counted. Psychological assessment tools for depression and anxiety, activities of daily living, and quality of life were not included in the extracted items for this review.

The ICF category codes contained in “mental functions (b110–b199)” were used to classify the domains of NPTs (Cieza et al., 2002). Cognitive assessments were identified as either full assessments, or subtests used as stand-alone assessments. The NPTs were categorized to the following purposes: (1) diagnostic, (2) screening. If the cognitive domains intended by the authors were different, the same assessment tools were organized by the cognitive areas intended by each study. That is, the same evaluation tool may be described in different cognitive domains.

The number of cases in each cognitive domain and the assessment tools in each domain was calculated as percentages. For all data pre-processing, agreement calculations, and figure creations, we used the statistical software JMP^®^ Pro (version 16.1.0) and statistical package R (version 4.1.2)^1^ (R Core Team, 2018).

## 3. Results

### 3.1. Literature search results

**FIGURE 1:**
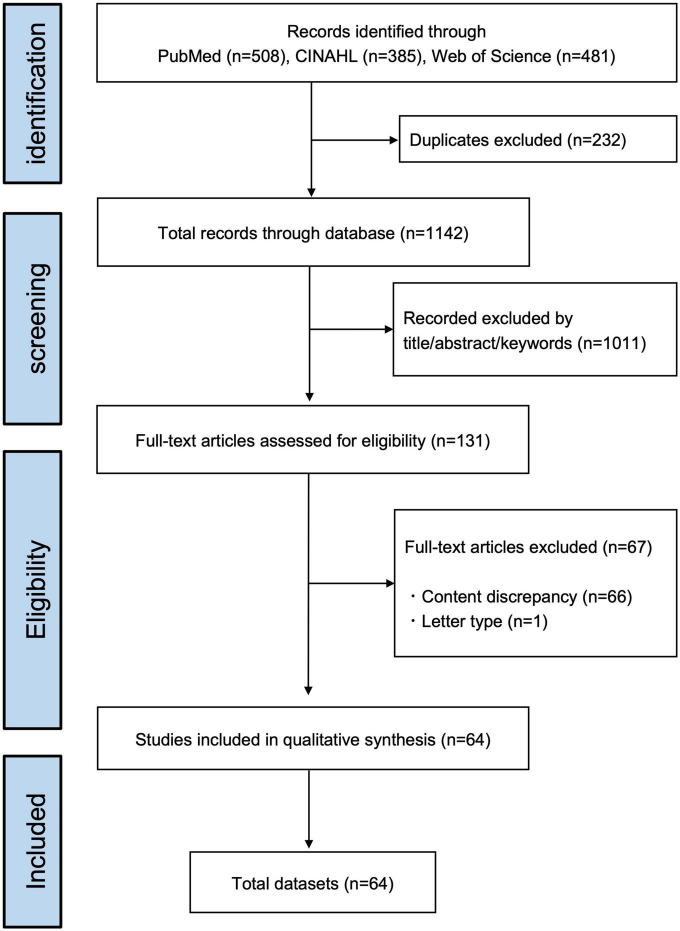
Flowchart of the literature search process.

The present study yielded a total of 1,374 records published through October 2021. After excluding duplicates (*n* = 232), 1,142 records remained. Independent screening by title, abstract, and keywords resulted in an initial 131 articles eligible for full-text papers. The information necessary for data charting was available without having to contact the authors. Reasons for exclusion criteria were publications of the content discrepancy (66 studies) and the publication type of letter (one study). After studying the eligibility, 64 papers were included by the two reviewers. The information data sets, which included all extracted data from each article, were summarized in Figure 1 and Supplementary Table 1 (table of characteristics of included studies with reference information).

### 3.2. Publication characteristics

#### 3.2.1. Years and regions of the publication

**FIGURE 2:**
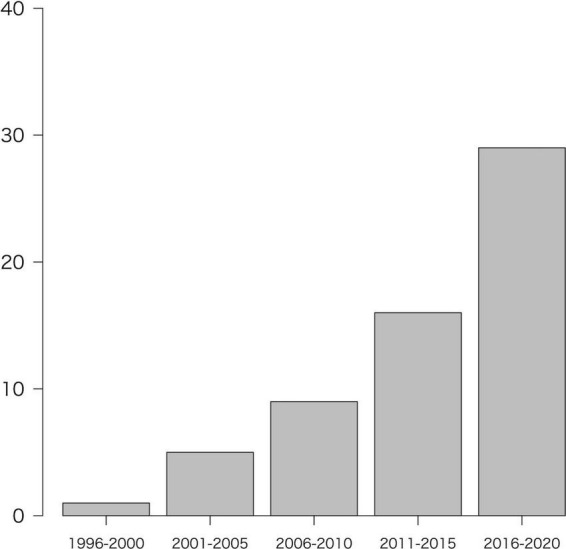
Number of included studies by year of publication. Note that the year 2021 (*n* = 4) was not included in the figure because it is less than 1 year.

The number of studies in the year of issue (divided into 5-year intervals) is shown in Figure 2. In order of most frequently reported, were North America (*n* = 22, 34.4%), Europe (*n* = 18; 28.1%), Asia (*n* = 18; 28.1%), Africa (*n* = 3, 4.7%), multi-region (*n* = 2, 3.1%), and South America (*n* = 1; 1.6%). The categories of regions were described in Supplementary material C.

#### 3.2.2. Study designs of publication

With respect to the publication type, 36 articles were longitudinal studies, and 28 articles were cross-sectional studies. Among them, 44 and 20 articles were single-center and multicenter studies, respectively.

#### 3.2.3. Sample size of publication

The total sample size at the baseline evaluation was distributed as follows: less than 50 (*n* = 32; 50.0%), 50–99 (*n* = 14; 21.9%), 100–699 (*n* = 17; 26.6%), and more than 700 (*n* = 1; 1.6%).

#### 3.2.4. Cancer types studied in publications

The most common types of cancer were breast cancer (*n* = 44; 68.8%), followed by colorectal cancer (*n* = 5; 7.8%), lymphoma (*n* = 2; 3.1%), testicular cancer (*n* = 2; 3.1%), ovarian cancer (*n* = 1; 1.6%), and lung cancer (*n* = 1; 1.6%). The percentage of studies that included different cancer types was 14% (*n* = 9).

### 3.3. Neuropsychological assessment tools (NPTs) and cognitive domains

Neuropsychological tests were classified according to the function intended by authors into the following domains: intellectual functions (b117), attention functions (b140), memory function (b144), psychomotor functions (b147), perceptual functions (b156), higher-level cognitive functions (b164), and mental function of language (b167). Those that did not match any of these categories were classified as mental functions, unspecified (b199). The classification procedure was based on the domains that were reported by the authors, and then the semantic categorized terms to the ICF domains were mapped using constant rules. For example, the semantic categories “verbal memory,” “visual memory” and “learning” were mapped to the memory functions domain. The semantic categories “executive function” and “cognitive flexibility” were mapped onto the higher-level cognitive functions domain (Please see Supplementary material D for other classification methods).

**FIGURE 3:**
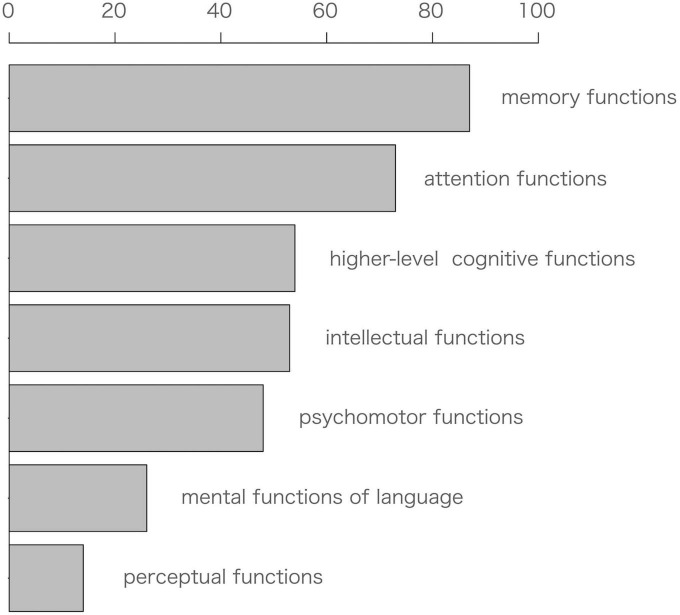
International Classification of Functioning, Disability and Health (ICF) domain sorting of neuropsychological tools. The ICF codes corresponding to the selected domains are as follows: b114 (memory functions), b140 (attention functions), b164 (higher-level cognitive functions), b117 (intellectual functions), b147 (psychomotor functions), b167 (mental functions of language), and b156 (perceptual functions).

Figure 3 showed the distribution of cognitive domains in the NPTs, with their results in the following order: memory functions were the most common (23.8%), followed by attention functions (20.0%), higher-level cognitive functions (14.8%), intellectual functions (14.5%), psychomotor functions (13.2%), mental functions of language (7.1%), and perceptual functions (3.4%).

**FIGURE 4:**
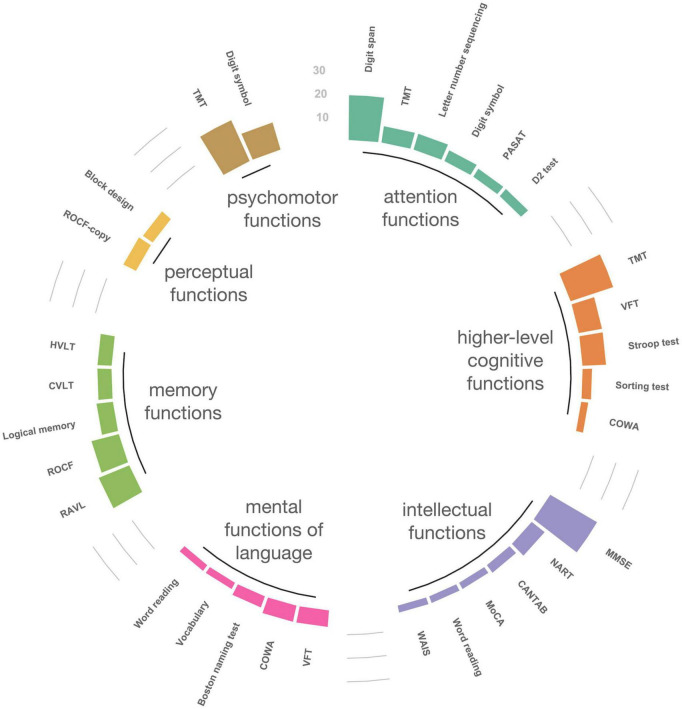
International Classification of Functioning, Disability and Health domain sorting map of neuropsychological tools. The top five tools in each domain were targeted, and those that were used only once or twice were excluded. TMT, trail making test; PASAT, paced auditory serial addition test; VFT, verbal fluency test; COWA, controlled oral word association test; MMSE, Mini-Mental State Examination; NART, National Adult Reading Test; CANTAB, Cambridge Neuropsychological Test Automated Battery; MoCA, Montreal Cognitive Assessment; WAIS, Wechsler Adult Intelligence Scale; RAVL, Rey Auditory Verbal Learning test; ROCF, Rey-Osterrieth Complex Figure test; CVLT, California Verbal Learning Test; HVLT, Hopkins Verbal Learning Test; Digit symbol, digit symbol substitution (coding) test. The following tools are subtests of the WAIS: letter number sequencing, vocabulary, block design. The word reading is a subtest of the Wide Range Achievement Test. Logical memory is a subtest of the Wechsler Memory Scale.

Figure 4 shows the map of the cognitive domains using an ICF framework. The top five tools in each domain were targeted, and those that were used only once or twice were excluded. Among the seven domains, the TMT was frequently used in three domains (attention; *n* = 7, psychomotor; *n* = 19, higher-level cognitive function; *n* = 19). The TMT-part A tool (the task of connecting numbers in sequence) was mainly used in the attention and psychomotor function domains, whereas the TMT-part B tool (the task that connects numbers and letters in alternating sequence) was mainly used in the higher-level cognitive function domain. The verbal fluency test (VFT) and the COWA were highly used in two domains: mental functions of language (VFT; *n* = 7, COWA; *n* = 7) and higher-level cognitive function (VFT; *n* = 10, COWA; *n* = 3) domains. Both tools assess word fluency, but in terms of the number of VFT and COWA used, COWA was less used in the higher-level cognitive function domain. These tools were mainly used to assess phonemic fluency (i.e., letter words) in the mental functions of language domain and as semantic fluency (i.e., animal words) in the higher-level cognitive function domains. Similarly, the Rey-Osterrieth Complex Figure test (ROCF) was used in both memory function (*n* = 12) and perceptual function (*n* = 6) domains. In the perceptual function domain, all used the ROCF-copy subtest, while in the memory functions domain, the ROCF-delayed recall subtest was the tool that was most used. Of the 64 studies included for the current study, 33 used the screening tools (eight used the screening tools only); 54 used the diagnosis tools (29 used the diagnosis tools only). In terms of screening tools, the Mini-Mental State Examination (MMSE) was used most frequently (*n* = 23) followed by the National Adult Reading Test (NART, *n* = 7).

### 3.4. Subjective cognitive tools/patient-reported outcomes (PROs)

Among PROs, the Functional Assessment of Cancer Therapy-Cognitive Function (FACT-Cog) was used in 14 studies, accounting for 56% of all PROs. Other PROs reported in two studies were the Patient Assessment of Own Functioning Inventory (PAOFI), the Cognitive Failures Questionnaire, the Everyday Cognition questionnaire, the Retrospective Memory and Prospective Memory questionnaires, and the Cognitive Problems in Daily Life.

### 3.5. Neuroimaging devices and measuring methods

Among neuroimaging devices, magnetic resonance imaging (MRI) was the most used (*n* = 19), followed by positron emission tomography (*n* = 2) and electroencephalography (*n* = 1). In the segment of MRI devices, structural and functional imaging evaluations were reported in ten and nine reports, respectively. Regarding measurement methods, seven studies consisted of the voxel-based morphometry (VBM) and three consisted of the diffusion tensor imaging (DTI) for structural MRI studies. Three studies were based on functional MRI (fMRI), and six studies on resting-state fMRI.

### 3.6. Association between the publishing year and the amount of tool use (including study design and sample size)

**FIGURE 5:**
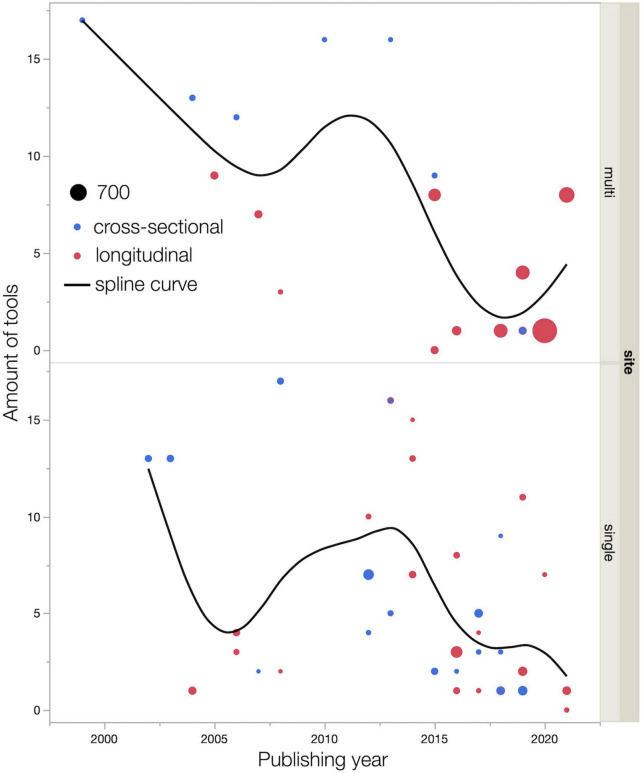
Relationships among publishing year, amount of tools use, and sample size. Dot size indicates sample size. Smoothing spline curves are used for estimating functional relationships between publishing year and amount of tools use. The smoothing parameter λ (lambda) is set at 0.05.

Figure 5 showed the association between the publishing year and the amount of tool use, including study design and sample size. In the early 2000s, both single-center and multicenter studies used more than 13 tools in a study. Around 2020, multicenter, longitudinal studies with large sample sizes were being conducted, and one to at most eight tools were used in the studies (Dhillon et al., 2018; Ng et al., 2018; Hormozi et al., 2019; Magnuson et al., 2019; Atallah et al., 2020; Van Dyk et al., 2021).

## 4. Discussion

In the present study, 36 longitudinal studies and 28 cross-sectional studies were systematically identified to investigate the assessment tools and cognitive domains used to assess CICIs, in accordance with PRISMA-ScR recommendations. Mapping the domains reported in studies using the ICF framework in NPTs revealed the extent of agreement between studies on the cognitive domains when each tool was used (Figure 4). The amount of tool use tended to diminish over the publication years. FACT-Cog was commonly used for PROs. Structural and functional studies using MRI together with brain imaging dominated the studies.

Regarding the validity of the extracted articles, recent review articles reported that between 17 and 101 cases of chemotherapy-induced cognitive dysfunction were extracted (Bray et al., 2018; Cerulla Torrente et al., 2020; Dijkshoorn et al., 2021). Because of differences in target disease and assessment tools, it would be difficult to make general comparisons. These studies extracted generally the same articles at the screening phase, and we believe that our search methodology is reasonable. In this study, we adopted studies that used prospective assessment tools to capture chemotherapy-induced cognitive dysfunction. Additionally, the exclusion of studies that did not distinguish between radiation or hormonal therapy was unique; the study by Li et al. (2018) used particularly ideal criteria. Hormonal therapy is particularly difficult to distinguish in the treatment process in breast cancer and prostate cancer. The present study did not include studies on hormone therapy, which may have led to the different eligibility selection of papers from other studies. However, considering the pathogenesis, a clear distinction should be recognized between hormonal therapy and chemotherapy (Mounier et al., 2020). On the other hand, it is noteworthy that breast cancer studies were the most frequently selected, even if hormonal therapy was omitted as much as possible. As a result, this review article may be as specific as possible to the assessment of chemotherapy-induced cognitive dysfunction. The scoping review by Vizer et al. (2022) should be referenced since it focused on studies on the Adolescent and Young Adult (AYA) generation, which was not the focus of the present review.

The most commonly used cognitive domain was memory functions, followed by attention functions, higher-level cognitive functions, and psychomotor functions. Perceptual function was relatively minimal. The results of the present study agreed with those of previous reviews of other domain classifications that have investigated the CRCI (Vardy et al., 2008; Wefel et al., 2011; Cerulla Torrente et al., 2020). Interestingly, the present study found that shared NPTs were not clearly identified in some ICF domains. The same NPTs were used among domains, as shown by the results in Figure 4. This indicates that it can be difficult to categorize a single NPT into a single cognitive domain, and it is worth noting that a single NPT can affect multiple cognitive domains. In the cognitive domains based on the ICF classification we used, the TMT covered three domains (attention, psychomotor, and higher-level cognitive function), while the VFT and COWA covered two cognitive domains, language and higher-level cognitive function. This indicates that most of the cognitive domains to investigate CRCI can be covered by using the three tools (TMT, COWA, HVLT-R) recommended by the ICCTF. In this respect, these NPTs recommended by the ICCTF are reasonable assessments, as they allow for a multi-dimensional view of chemotherapy-induced cognitive functions. Meanwhile, the present review indicates that fewer HVLT-R and COWA were used compared to TMTs. The HVLT-R has similar concepts of the Rey Auditory Verbal Learning test (RAVLT), and the COWA evaluates the same concept as the VFT. Thus, although the names of the NPTs are different, there are cases where the concept to be evaluated is the same. This may be more likely to occur because of language-mediated procedures. Compared to NPTs that do not rely as much on language, NPTs that use language are more difficult to translate and adapt across languages and cultures, resulting in different NPTs with the same concept.

As Figures 2, 5 shows, the number of reports increase over the years, but sample sizes for both cross-sectional and longitudinal studies are generally small. This indicates that, as with CRCI, the research trend on CICIs has attracted interest, but the studies revealed a trend of poor quality of study design. Figure 5 shows that the number of tools used tends to gradually decrease over the years for both single and multicenter studies. Our study indicates that in the early 2000s, more than 13 tools were used to identify CICIs in exploratory research. We also believe that in the 2020s, the number of multicenter longitudinal studies have increased, and the number of tools has decreased. This indicates a trend toward a clear understanding of the NPTs and cognitive domains that need more attention for the CICIs, which was explored by studies in the 2000s. Recent reports have used tools that can assess multiple domains with a single tool, such as the Cambridge Neuropsychological Test Automated Battery (CANTAB) (Vardy et al., 2015; Magnuson et al., 2016; Dhillon et al., 2018; Williams et al., 2018). The results indicate that these personal computer-based assessment batteries are a standardized, multidisciplinary, well-developed single tool that could be widely used in future studies of the CICIs.

Different chemotherapeutics and cancer types may influence different cognitive domains. Some review articles suggest that different chemotherapeutics and cancer types may influence different cognitive domains (Subramaniam et al., 2020; Orszaghova et al., 2021). A previous study of breast cancer survivors reported that taxanes, compared to other cognitive domains, affected attention, psychomotor speed, and memory function (Cerulla et al., 2017). One study of breast cancer survivors with anthracyclines reported effects on verbal memory function using HVLT-R (Kesler and Blayney, 2016). Consecutive colorectal cancer patients receiving 5-fluorouracil and oxaliplatin-based adjuvant chemotherapy showed impaired higher-level cognitive function (executive function), including the VFT, compared to other cognitive domains (Sales et al., 2019). On the level of the biological control, the mechanisms of CICIs are mediated by a variety of factors including: direct neurotoxicity, inhibition of hippocampal neurogenesis, white and gray matter reduction, oxidative stress response, reduced cerebral blood flow, blood-brain barrier damage, neuroinflammation, hormonal changes, decreased hypothalamic-pituitary-adrenal axis activity, and microbiota-gut-brain axis dysfunction (Mounier et al., 2020; Subramaniam et al., 2020; Orszaghova et al., 2021). In recent years, chronic inflammation and neuroinflammatory pathways have been considered the main factors of CICIs. Inflammation-related problems have been shown to be closely related to attentional function in the cognitive domains (Oppegaard et al., 2021). The frequent use of attention domain tools in our current review may show that many researchers in the field support the idea of inflammation-induced attention deficit.

Neuroimaging assists in a more accurate understanding of the NPT results. In a PET study examining different brain activity areas during short-term memory tasks, the most significant difference between the chemotherapy and control groups was in the inferior frontal gyrus (Silverman et al., 2007). MRI-VBM analysis showed decreased gray matter density in the inferior and middle frontal gyrus and cerebellum in breast cancer survivors after chemotherapy. Furthermore, the decrease in gray matter density in the right middle frontal gyrus was related to the dose of chemotherapy for VFT (Li et al., 2018). In other words, decreased activity in the prefrontal cortex may be important for capturing the symptoms of the CICIs. These neuroimaging studies have the potential to identify specific cognitive domains that should be targeted by the NPTs. However, while PET and MRI-based studies are useful in that they can reveal structural brain changes and functional brain changes, they are likely to be costly and unsuitable for general use in clinical care. To address this issue, we anticipate the possibility of adopting portable EEG and near-infrared spectroscopy (NIRS) instruments that are less expensive and constrained in the future to aid in diagnosis as a way to overcome these problems (Jean-Pierre, 2014; Suhaimi et al., 2020).

### 4.1. Strengths and limitations

This scoping review reported cognitive assessment tools used to assess CICIs in the CRCI. To this end, a cognitive assessment tool for cancer in the domains of the ICF framework was summarized by peer review using the chart method. The ICF framework was used for the international consensus. In recent years, it has also been applied to scoping reviews for the assessment of cognitive function after a stroke (Saa et al., 2019). Furthermore, this study has the unique advantage of examining the trends in reporting not only NPTs, but also PROs and neuroimaging devices for CICIs. However, this scoping review has several limitations.

The main limitation of the present study is that we summarized several reports on different diseases and chemotherapeutic agents rather than focusing on one agent. Second, we did not investigate the influences of cancer-induced fatigue, anxiety, and depression on cognitive function. Other factors such as age, educational history, hormone levels, sleep disturbances, and anemia are also likely to influence cognitive function (Lange et al., 2019; Orszaghova et al., 2021). Therefore, in the future, more rigorous studies controlling for the effects of confounding factors are warranted. Regarding the charting method, this review does not include gray literature. Although gray literature is an important source of information, this study targeted peer-reviewed articles with standard quality assurance of content. Furthermore, we did not assess the quality of the methodology for the selected studies. The review articles are biased toward the female gender as most studies were those of breast cancer patients or were focused on breast cancer. It would be desirable to see more reports on target populations other than breast cancer patients in the future.

## 5. Conclusion

Interest in the CICIs has increased over time, and the cognitive assessment tools used were mapped in this study. The memory and attention functions were found to be common ICF domains in the NPT. There was a gap between commonly recommended NPTs and tools employed in the study. For PROs, the FACT-Cog was clearly the shared tool used. We believe that integrating assessment tools for CRCIs from the perspective of the ICF domains can address interdisciplinary issues. This research is crucial for multidisciplinary care and rehabilitation treatments for cancer survivors with CICIs.

## Author contributions

KS and SA performed the material preparation, data collection, and analysis. KS wrote the first draft of the manuscript. All authors commented on previous versions of the manuscript, contributed to the study conception and design, and read and approved the final manuscript.

## Footnotes

1
http://www.r-project.org
