# Supplementary material for: A scoping review of cognitive assessment tools and domains for chemotherapy-induced cognitive impairments in cancer survivors
Source: Front Hum Neurosci. 2023 Feb 20;17:1063674. doi: 10.3389/fnhum.2023.1063674 (PMC9987518; doi:10.3389/fnhum.2023.1063674)
Supplement: Supplementary file 2 [file Data_Sheet_2.docx]

| **MeSH terms** | **Cognition Disorders** |
| --- | --- |
| **Synonym** | Disorder, Cognition; Diorders, Cognition |
| **MeSH terms found below this term in the MeSH hierarchy** | Cognitve Dysfunction |
| **Synonym** | Cognitive Dysfunctions; Dysfunction, Cognitive; Dysfunctions, Cognitive  Cognitive Impairments; Cognitive Impairment; Impairment, Cognitive; Impairments, Cognitive  Mild Cognitive Impairment; Cognitive Impairment, Mild; Cognitive Impairments, Mild; Impairment, Mild Cognitive  ; Impairments, Mild Cognitive; Mild Cognitive Impairments  Mild Neurocognitive Disorder; Disorder, Mild Neurocognitive; Disorders, Mild Neurocognitive; Mild Neurocognitive Disorders  Neurocognitive Disorder, Mild; Neurocognitive Disorders, Mild  Cognitive Decline; Cognitive Declines; Decline, Cognitive; Declines, Cognitive  Mental Deterioration; Deterioration, Mental; Deteriorations, Mental; Mental Deteriorations |
| **MeSH terms found below this term in the MeSH hierarchy** | Chemotherapy-Related Cognitive Impairment |
| **Synonym** | Chemotherapy Related Cognitive Impairment; Chemotherapy-Related Cognitive Impairments; Cognitive Impairment, Chemotherapy-Related; Cognitive Impairments, Chemotherapy-Related  Chemotherapy-Induced Cognitive Dysfunction; Chemotherapy Induced Cognitive Dysfunction; Cognitive Dysfunction, Chemotherapy-Induced  Chemo-Fog; Chemo Fog  Chemotherapy-Induced Cognitive Impairments; Chemotherapy Induced Cognitive Impairments; Cognitive Impairment, Chemotherapy-Induced; Cognitive Impairments, Chemotherapy-Induced; Chemotherapy-Induced Cognitive Impairment; Chemotherapy Induced Cognitive Impairment  Chemotherapy-Related Cognitive Dysfunction; Chemotherapy Related Cognitive Dysfunction; Chemotherapy-Related Cognitive Dysfunctions; Cognitive Dysfunction, Chemotherapy-Related  Chemobrain |

**Supplementary Material B: MeSH terms and synonyms that are below this term in the MeSH hierarchy**

| **MeSH terms** | **Neoplasms** |
| --- | --- |
| **Synonym** | Tumor; Tumors  Neoplasm; Neoplasia; Neoplasias  Cancer; Cancers  Malignant Neoplasm; Malignant Neoplasms; Neoplasm, Malignant; Neoplasms, Malignant  Malignancy; Malignancies  Benign Neoplasms; Benign Neoplasm; Neoplasms, Benign; Neoplasm, Benign |

| **MeSH terms** | **Drug Therapy** |
| --- | --- |
| **Synonym** | Therapy, Drug; Drug Therapies; Therapies, Drug  Chemotherapy; Chemotherapies  Pharmacotherapy; Pharmacotherapies |
| **Typical MeSH terms found below this term in the MeSH hierarchy** | **Chemotherapy, Adjuvant** |
| **Typical Synonyms** | Adjuvant Drug Therapy; Drug Therapy, Adjuvant; Adjuvant Chemotherapy |
