# Supplementary material for: A scoping review of cognitive assessment tools and domains for chemotherapy-induced cognitive impairments in cancer survivors
Source: Front Hum Neurosci. 2023 Feb 20;17:1063674. doi: 10.3389/fnhum.2023.1063674 (PMC9987518; doi:10.3389/fnhum.2023.1063674)
Supplement: Supplementary file 3 [file Data_Sheet_3.docx]

| **Region** | **Country** | **Number of studies** |
| --- | --- | --- |
| North America | United States | 18 |
|  | Canada | 4 |
| Asia | People's Republic of China | 10 |
|  | Singapore | 3 |
|  | Iran | 1 |
|  | India | 1 |
|  | Japan | 1 |
|  | South Korea | 1 |
|  | Taiwan | 1 |
| Europe | Netherlands | 4 |
|  | Italy | 4 |
|  | Germany | 3 |
|  | France | 2 |
|  | Spain | 2 |
|  | Greece | 1 |
|  | Poland | 1 |
|  | United Kingdom | 1 |
| Africa | Egypt | 1 |
|  | Ethiopia | 1 |
|  | South Africa | 1 |
| Multiple | Canada and Australia | 2 |
| South America | Brazil | 1 |

Supplementary Material C. Categories of regions/countries and number of studies
