# Supplementary material for: A scoping review of cognitive assessment tools and domains for chemotherapy-induced cognitive impairments in cancer survivors
Source: Front Hum Neurosci. 2023 Feb 20;17:1063674. doi: 10.3389/fnhum.2023.1063674 (PMC9987518; doi:10.3389/fnhum.2023.1063674)
Supplement: Supplementary file 4 [file Data_Sheet_4.docx]

Supplementary Material D. Definition and classification of cognitive domains

| **Cognitive domains** | **ICF code** |  | **Included studies No. (#1–64)** |
| --- | --- | --- | --- |
| **intellectual functions** | b117 | intelligence | #54 |
|  |  | intelligence and mental status | #46 |
|  |  | intelligence and general cognitive functioning | #63 |
|  |  | global intelligence | #11 |
|  |  | premorbid intelligence | #11, #25 |
|  |  | premorbid intelligence level | #52 |
|  |  | premorbid intelligence quotient | #43 |
|  |  | pre-morbid verbal intelligence | #50, #51 |
|  |  | estimated premorbid IQ | #55 |
|  |  | estimated IQ | #56, #57 |
|  |  | nonverbal intellectual capacity | #43 |
|  |  | cognitive function | #1, #38, #41 |
|  |  | cognitive performance | #26 |
|  |  | cognitive impairment | #1, #5, #48 |
|  |  | general cognitive functions | #15–17, #37, #40 |
|  |  | global cognitive function | #11, #60, #63 |
|  |  | normal cognitive function | #36, #58 |
|  |  | objective cognitive function | #39, #47 |
|  |  | screening | #33, #59 |
|  |  | brief cognitive screening tool | #42 |
|  |  | brief screening test for cognitive deficits | #27 |
|  |  | dementia screener | #32 |
|  |  | screened for dementia | #24, #49 |
|  |  | neuropsychological screening battery | #28 |
|  |  | screening tool to assess cognitive dysfunction | #53 |
|  |  | neuropsychological assessments (attention and simple reaction time, discriminability memory, verbal learning and memory, spatial working memory, attention and complex, reaction time, discriminability learning) | #23 |
|  |  | cognitive subdomains, including executive function and attention, episodic memory, working memory, and language processing | #14 |
|  |  | several domains (attention, memory, language, frontal functions) | #9 |
|  |  | visual memory, executive functioning, attention, verbal memory, and cognitive processing | #62 |
|  |  | seven measures that target the subdomains of executive function, episodic memory, language, processing speed, working memory, and attention. | #13 |
|  |  | cognitive function in 4 domains (processing speed, response speed, memory, attention) | #44, #45 |
| **attention functions** | b140 | attention | #9, #39, #42, #49, #60, #61, #63, #64 |
|  |  | attention (vigilance, accuracy) | #2, #3 |
|  |  | attentional function | #7 |
|  |  | alertness | #52 |
|  |  | attention/concentration | #50, #51 |
|  |  | focused and sustained attention | #57 |
|  |  | concentration | #25 |
|  |  | sustain attention | #31 |
|  |  | sustained attention and short-term memory | #11 |
|  |  | attention and memory | #24 |
|  |  | attention and working memory | #21, #46, #56 |
|  |  | working memory | #4, #19, #34, #35, #37, #48, #54 |
|  |  | visuo-spatial working memory | #52 |
|  |  | verbal working memory | #25, #52 |
|  |  | attention, verbal short-term memory | #25 |
|  |  | selective attention | #52 |
|  |  | attention and visual-motor ability | #22 |
|  |  | visual-selective attention | #11 |
|  |  | attention reaction time (reaction time) | #2–4 |
|  |  | reaction time | #12 |
|  |  | distractibility, reaction time | #2 |
| **memory functions** | b144 | memory | #9, #20, #46, #49, #63 |
|  |  | memory ability | #42 |
|  |  | memory tests | #8 |
|  |  | memory function | #15–17, #30 |
|  |  | short-term memory | #36 |
|  |  | episodic memory, verbal | #34, #48 |
|  |  | episodic memory, visual | #34, #48 |
|  |  | short- and long-term skill | #11 |
|  |  | verbal memory | #2–6, #12, #19, #21, #22, #25, #35, #39, #50, #51, #54, #55–57 |
|  |  | visual memory | #2–4, #12, #19, #35, #50, #51, #54, #56, #57 |
|  |  | visuo-spatial memory | #5 |
|  |  | visual delayed recall, memory | #52 |
|  |  | learning | #20 |
|  |  | verbal learning | #2, #3, #11, #12, #23 |
|  |  | verbal learning and memory | #10, #32, #52, #59, #61 |
|  |  | capacity of verbal and nonverbal learning functions | #4 |
| **psychomotor functions** | b147 | psychomotor domain | #20 |
|  |  | psychomotor function | #2, #3 |
|  |  | psychomotor task, attention | #63 |
|  |  | psychomotor function, selective attention | #25 |
|  |  | psychomotor speed | #12, #46, #61 |
|  |  | psychomotor function, information processing speed | #25 |
|  |  | processing speed | #4, #11, #19, #32, #34 |
|  |  | processing speed and vigilance | #54 |
|  |  | information processing speed | #5 |
|  |  | speed of information processing | #50–52 |
| **perceptual functions** | b156 | spatial ability | #2, #3 |
|  |  | visuospatial function | #12, #46, #50, #51, #63 |
|  |  | visuospatial domain | #20 |
|  |  | visuospatial ability | #32, #56 |
| **mental function of language** | b167 | language | #8, #20, #46, #56, #63 |
|  |  | language/fluency | #9 |
|  |  | the ability to find words from the internal lexicon | #11 |
|  |  | verbal ability | #2–4 |
|  |  | verbal fluency | #12, #39 |
|  |  | verbal function/functioning | #50, #51, #57 |
| **higher-level cognitive functions** | b164 | executive function | #21, #22, #25, #27, #34, #37, #40, #46, #48, #49, #52, #54, #56, #58, #59, #61, #63 |
|  |  | executive domain | #20 |
|  |  | executive attention | #12 |
|  |  | executive function and cognitive flexibility | #31 |
|  |  | cognitive flexibility, psychomotor function, divided attention, | #25 |
|  |  | cognitive flexibility, divided attention | #52 |
|  |  | mental flexibility | #11, #50, #51, #57 |
|  |  | processing speed and inhibition as an element of executive function | #32 |
|  |  | verbal fluency (executive function) | #32, #56 |
|  |  | sorting | #2 |
|  |  | thinking and reasoning | #24 |
| **mental functions, unspecified** | b199 | motor functioning/function | #2, #3, #28, #50, #51, #61 |
|  |  | motor speed | #57 |
|  |  | Motor speed and dexterity | #32, #57 |
