# Supplementary material for: A scoping review of cognitive assessment tools and domains for chemotherapy-induced cognitive impairments in cancer survivors
Source: Front Hum Neurosci. 2023 Feb 20;17:1063674. doi: 10.3389/fnhum.2023.1063674 (PMC9987518; doi:10.3389/fnhum.2023.1063674)
Supplement: Supplementary file 5 [file Table_1.pdf]

Table 1. Characteristics of included studies

**No.**	**First author/ Year**	**Continent /Country**	**Study design**		**Sample size**	**Cancer type**	**Assessment tools [cognitive domain]**<br>**For screening**	**For diagnosis**	**PROs**	**Neuroimaging tools**
**1**	(Abebe et al., 2021)	**Africa** /Ethiopia	longitudinal	single	**C+: 117**<br>C-: 117	breast	MMSE **[intellectual functions: b117]**	none	none	none
2	(Ahles et al., 2002)	**North America** /United States	cross-sectional	single	**C+: 71**<br>(35/36)<br>C-: 57<br>(35/22)	breast, lymphoma	none	Vocabulary (WAIS-III) **[mental functions of language: b167]**<br>Reading subtest (WRAT-III) **[mental functions of language: b167]**<br>Boston naming test **[mental functions of language: b167]**<br>COWA **[mental functions of language: b167]**<br>Block design (WAIS-III) **[perceptual functions: b156]**<br>CVLT **[memory functions: b144]**<br>Logical memory (WMS-R) **[memory functions: b144]**<br>Visual reproduction (WMS-R) **[memory functions: b144]**<br>Digit symbol (WAIS III) **[psychomotor functions: b147]**<br>TMT-A/B **[psychomotor functions: b147]**<br>Finger tapping **[mental functions, unspecified: b199]**<br>Thumb-finger15 sequencing **[mental functions, unspecified: b199]**<br>CPT **[attention functions: b140]**	none	none
**3**	(Ahles et al., 2003)	**North America** **/**United States	cross-sectional	single	**C+: 80**<br>(51/29)	breast, lymphoma	none	Vocabulary (WAIS III) **[mental functions of language: b167]**<br>Reading subtest (WRAT-III) **[mental functions of language: b167]**<br>Boston naming test **[mental functions of language: b167]**<br>COWA **[mental functions of language: b167]**<br>Block design (WAIS-III) **[perceptual functions: b156]**<br>CVLT **[memory functions: b144]**<br>Logical memory (WMS-R) **[memory functions: b144]**<br>Visual reproduction (WMS-R) **[memory functions: b144]**<br>Digit symbol (WAIS-III) **[psychomotor functions: b147]**<br>TMT-A/B **[psychomotor functions: b147]**<br>Finger tapping **[mental functions, unspecified: b199]**<br>Thumb-finger15 sequencing **[mental functions, unspecified: b199]**<br>CPT **[attention functions: b140]**	none	none

**4**	(Ahles et al., 2014)	**North America** /United States	longitudinal	single	**C+: 55**<br>C-: 68<br>HC: 43	breast	none	Vocabulary (WAIS-III) **[mental functions of language: b167]**<br>VFT (D-KEFS) **[mental functions of language: b167]**<br>CVLT **[memory functions: b144]**<br>Logical memory (WMS-III) **[memory functions: b144]**<br>Visual reproduction (WMS-R) **[memory functions: b144]**<br>PASAT **[attention functions: b140]**<br>Digit symbol (WAIS-III) **[psychomotor functions: b147]**<br>TMT (D-KEFS) **[psychomotor functions: b147]**<br>CWIT (D-KEFS) **[psychomotor functions: b147]**<br>Grooved pegboard **[psychomotor functions: b147]**<br>Sorting test (D-KEFS) **[higher-level cognitive functions: b164]**<br>CPT **[attention functions: b140]**<br>Block design (WAIS III) **[perceptual functions: b156]**	none	none
**5**	(Andreis et al., 2013)	**Europe** /Italy	longitudinal	single	**C+: 47**	colon	MMSE **[intellectual functions: b117]**	Clock drawing test **[memory functions: b144]**<br>ROCF **[memory functions: b144]**<br>TMT-A/B **[psychomotor functions: b147]**<br>RAVLT **[memory functions: b144]**	none	none
**6**	(Andryszak et al., 2018)	**Europe** /Poland	longitudinal	single	**C+: 31**<br>HC: 30	breast	none	RAVLT **[memory functions: b144]**	none	none
**7**	(Atallah et al., 2020)	**North America** /United States	longitudinal	multi	**C+: 1329**	breast, gastrointestinal, gynecological, or lung	none	16-item ver. AFI **[attention functions: b140]**	none	none
**8**	(Bai et al., 2021)	**Asia** /People's Republic of China	cross-sectional	single	**C+: 19**<br>C-: 11	breast	none	AVLT **[memory functions: b144]**	FACT-Cog	rs-fMRI<br>MRI-DTI
**9**	(Baudino et al., 2012)	**Europe** /Italy	cross-sectional	single	**C+:32**<br>C-:18	lymphoma	MMSE **[intellectual functions: b117]**	TMT-B **[attention functions: b140]**<br>Phonemic fluency **[mental functions of language: b167]**<br>Short story test **[memory functions: b144]**	none	PET
**10**	(Bender et al., 2006)	**North America** /United States	longitudinal	single	**C+: 46**	breast	none	RAVLT **[memory functions: b144]**<br>ROCF **[memory functions: b144]**<br>Four-word short memory test **[memory functions: b144]**	PAOFI	none

**11**	(Biglia et al., 2012)	**Europe** /Italy	longitudinal	single	**<u>C+: 40</u>**	breast	NART **[intellectual functions: b117]**<br>MMSE **[intellectual functions: b117]**	Attentive matrices **[attention functions: b140]**<br>Digit span- forward **[attention functions: b140]**<br>TMT-A **[psychomotor functions: b147]**<br>TMT-B **[higher-level cognitive functions: b164]**<br>Phonemic word fluency **[mental functions of language: b167]**<br>Short story **[memory functions: b144]**<br>RAVLT **[memory functions: b144]**<br>RCPM **[intellectual functions: b117]**	FACT-Cog	none
**12**	(Castellon et al., 2004)	**North America /**United States	cross-sectional	multi	**<u>C+: 53</u>**<br>HC: 19	breast	none	COWA **[mental functions of language: b167]**<br>Animal fluency **[mental functions of language: b167]**<br>Logical memory **[memory functions: b144]**<br>CVLT **[memory functions: b144]**<br>Visual reproduction (WMS-R) **[memory functions: b144]**<br>ROCF-recall **[memory functions: b144]**<br>Block design **[perceptual functions: b156]**<br>ROCF-copy **[perceptual functions: b156]**<br>Digit symbol **[psychomotor functions: b147]**<br>TMT-A/B **[psychomotor functions: b147]**<br>CalCAP [**[attention functions: b140]**]<br>PASAT **[higher-level cognitive functions: b164]**<br>Stroop interference **[higher-level cognitive functions: b164]**	CFQ	none
**13**	(Chen et al., 2018a)	**North America /**United States	longitudinal	single	**<u>C+: 16</u>**<br>HC: 14	breast	none	the NIH Toolbox for Cognition **[intellectual functions: b117]**	none	MRI-VBM
**14**	(Chen et al., 2018b)	**North America /**United States	longitudinal	single	**<u>C+:14</u>**<br>HC: 13	breast	none	the NIH Toolbox for Cognition **[intellectual functions: b117]**	none	MRI-VBM
**15**	(Cheng et al., 2013)	**Asia** /People's Republic of China	cross-sectional	single	**<u>C+: 40</u>**<br>HC: 40	breast	MMSE **[intellectual functions: b117]**	VFT **[memory functions: b144]**<br>Digit span **[memory functions: b144]**<br>EPM **[memory functions: b144]**<br>TPM **[memory functions: b144]**	none	none
**16**	(Cheng et al., 2016)	**Asia** /People's Republic of China	longitudinal	single	**<u>C+: 245</u>**	breast	MMSE **[intellectual functions: b117]**	VFT **[memory functions: b144]**<br>Digit span **[memory functions: b144]**	RMQ/PMQ	none
**17**	(Cheng et al., 2017)	**Asia** /People's Republic of China	cross-sectional	single	**<u>C+: 34</u>**<br>HC: 31	breast	MMSE **[intellectual functions: b117]**	VFT **[memory functions: b144]**<br>Digit span **[memory functions: b144]**<br>EPM **[memory functions: b144]**<br>TPM **[memory functions: b144]**	none	rs-fMRI

**18**	(Cheung et al., 2015)	**Asia** /Singapore	longitudinal	multi	**C+: 99**	breast	none	None	FACT-Cog	none
**19**	(Collins et al., 2013)	**North America /**Canada	longitudinal	single	**C+: 60**<br>HC: 60	breast	none	Digit symbol (WAIS-III) **[psychomotor functions: b147]**<br>Symbol search (WAIS-III) **[psychomotor functions: b147]**<br>TMT-A/B **[psychomotor functions: b147]**<br>Processing speed index (CNS-VS) **[psychomotor functions: b147]**<br>Reaction time index (CNS-VS) **[psychomotor functions: b147]**<br>Digit Span (WAIS-III) **[attention functions: b140]**<br>Letter Number Sequencing (WAIS-III) **[attention functions: b140]**<br>PASAT **[attention functions: b140]**<br>CCCs **[attention functions: b140]**<br>COWA **[attention functions: b140]**<br>Flexibility index (CNS-VS) **[attention functions: b140]**<br>Working memory index (CNS-VS) **[attention functions: b140]**<br>HVLT-R **[memory functions: b144]**<br>BVMT-R **[memory functions: b144]**<br>Verbal memory index (CNS-VS) **[memory functions: b144]**<br>Visual memory index (CNS-VS) **[memory functions: b144]**	none	none
**20**	(Conroy et al., 2013)	**North America /**United States	cross-sectional	single	**C+: 24**<br>HC: 23	breast	none	RAVLT **[memory functions: b144]**<br>Brown learning test **[memory functions: b144]**<br>Digit span (WAIS-III) **[attention functions: b140]**<br>PASAT **[attention functions: b140]**<br>Reading subtest（WRAT-IV）**[mental functions of language: b167]**<br>Vocabulary (WAIS) **[mental functions of language: b167]**<br>Block design (WAIS) **[perceptual functions: b156]**<br>Digit span- backward **[higher-level cognitive functions: b164]**<br>COWA **[higher-level cognitive functions: b164]**<br>CWIT- inhibition and inhibition/switching (D-KEFS) **[higher-level cognitive functions: b164]**<br>Sorting test (D-KEFS) **[higher-level cognitive functions: b164]**<br>TMT-B (D-KEFS) **[higher-level cognitive functions: b164]**<br>CWIT- color naming (D-KEFS) **[psychomotor function: b147]**<br>TMT-A (D-KEFS) **[psychomotor functions: b147]**<br>Symbol digit modalities test **[psychomotor functions: b147]**<br>Grooved pegboard **[psychomotor functions: b147]**	FACT-COG<br>MASQ	fMRI<br>MRI-VBM
**21**	(Correa et al., 2017)	**North America /**United States	cross-sectional	single	**C+: 18**<br>HC: 18	ovarian, peritoneal and fallopian tube	none	BTA **[attention functions: b140]**<br>Symbol span (WMS-IV) **[attention functions: b140]**<br>TMT-A/B **[higher-level cognitive functions: b164)]**<br>COWA **[higher-level cognitive functions: b164]**<br>CVLT **[memory functions: b144]**	none	fMRI<br>MRI-VBM
**22**	(Cruzado et al., 2014)	**Europe** /Spain	longitudinal	single	**C+: 81**	colorectal	none	TMT-A **[attention functions: b140]**<br>Digit symbol (WAIS-R) **[attention functions: b140]**<br>Stroop **[higher-level cognitive functions: b164]**<br>TMT-B **[higher-level cognitive functions: b164]**<br>Verbal memory subtest of the Barcelona test **[memory functions: b144]**<br>Luria memory words test **[memory functions: b144]**<br>Spanish adaptation Barcelona test **[memory functions: b144]**	none	none

								Cognitive tools and domains for CICI		
**23**	(Dhillon et al., 2018)	**Multi** /Canada and Australia	longitudinal	multi	**C+: 362**<br>HC: 72	colorectal	none	CANTAB **[intellectual functions: b117]**	FACT-Cog	none
**24**	(Eberhardt et al., 2006)	**Europe** /Germany	longitudinal	single	**C+: 77**	Malignant hematological diseases or cancer of the gastrointestinal tract	Dementia test **[intellectual functions: b117]**	SKT **[attention functions: b140]**<br>Verbal and nonverbal learning tests **[memory function: b144]**<br>LPS **[higher-level cognitive functions: b164]**	none	none
**25**	(Hermelink et al., 2007)	**Europe** /Germany	longitudinal	multi	**C+: 101**	breast	none	Logical memory (WMS-R) **[memory functions: b144]**<br>Digit span (WMS-R) **[attention functions: b140]**<br>Digit symbol (WAIS) **[psychomotor functions: b147]**<br>D2 test **[attention functions: b140]**<br>TMT-A **[psychomotor functions: b147]**<br>TMT-B **[higher-level cognitive functions: b164]**<br>Word Fluency Test **[higher-level cognitive functions: b164]**<br>MWT-B **[intellectual functions: b117]**	none	none
**26**	(Hormozi et al., 2019)	**Asia** /Iran	longitudinal	multi	**C+: 100**	breast	MMSE **[intellectual functions: b117]**	none	none	none
**27**	(Iconomou et al., 2004)	**Europe** /Greece	longitudinal	single	**C+: 102**	lung, breast, colorectal, genitourinary, and other	MMSE **[intellectual functions: b117]**	none	none	none
**28**	(Jansen et al., 2008)	**North America /**United States	longitudinal	multi	**C+: 30**	breast	none	RBANS **[intellectual functions: b117]**<br>Stroop **[higher-level cognitive functions: b164]**<br>Grooved pegboard **[mental functions, unspecified: b199]**	none	none
**29**	(Keetile et al., 2021)	**Africa /** South Africa	longitudinal	single	**C+: 30**	breast	none	none	FACT-Cog	none
**30**	(Khan et al., 2016)	**Asia** /India	longitudinal	single	**C+: 68**	lymphoma	MMSE **[intellectual functions: b117]**	none	none	none
**31**	(Khan et al., 2019)	**North America /**Canada	longitudinal	single	**C+: 142**	breast and colorectal	none	PVT **[attention functions: b140]**<br>TMT-B **[higher-level cognitive functions: b164]**	none	none

**32**	(Koppelmans et al., 2012)	**Europe** /Netherlands	cross-sectional	single	**C+: 196**<br>C-: 1509	breast	MMSE **[intellectual functions: b117]**	15-WLT **[memory functions: b144]**<br>LDST **[psychomotor functions: b147]**<br>Stroop **[higher-level cognitive functions: b164]**<br>WFT **[higher-level cognitive functions: b164]**<br>DOT **[perceptual functions: b156]**<br>PPB **[mental functions, unspecified: b199]**	none	none
**33**	(Kotb et al., 2019)	**Africa** / Egypt	cross-sectional	single	**C+: 150**	hematological malignancy	MoCA **[intellectual functions: b117]**	none	none	none
**34**	(Lange et al., 2016)	**Europe** /France	longitudinal	single	**C+: 58**<br>CT-: 61<br>HC: 62	breast	none	G & B test **[memory functions: b144]**<br>ROCF **[memory functions: b144]**<br>Arithmetic (WAIS-III) **[attention functions: b140]**<br>Digit Span (WAIS-III) **[attention functions: b140]**<br>Letter-number sequencing (WAIS-III) **[attention functions: b140]**<br>TMT-A **[psychomotor functions: b147]**<br>TMT-B **[higher-level cognitive functions: b164]**<br>VFT **[higher-level cognitive functions: b164]**	FACT-Cog	none
**35**	(Lepage et al., 2014)	**North America** /Canada	longitudinal	single	**C+: 19**<br>HC: 19	breast	none	Digit symbol (WAIS-III) **[psychomotor functions: b147]**<br>Symbol search (WAIS-III) **[psychomotor functions: b147]**<br>TMT-A/B **[psychomotor functions: b147]**<br>Processing speed index (CNS-VS) **[psychomotor functions: b147]**<br>Digit span (WAIS-III) **[attention functions: b140]**<br>Letter number sequencing (WAIS-III) **[attention functions: b140]**<br>PASAT **[attention functions: b140]**<br>CCCs **[attention functions: b140]**<br>COWA **[attention functions: b140]**<br>Flexibility index (CNS-VS) **[attention functions: b140]**<br>Working memory index (CNS-VS) **[attention functions: b140]**<br>HVLT-R **[memory functions: b144]**<br>Verbal memory index (CNS-VS) **[memory functions: b144]**<br>BVMT-R **[memory functions: b144]**<br>Visual memory index (CNS-VS) **[memory functions: b144]**	none	MRI-VBM
**36**	(Li et al., 2017)	**Asia** /People's Republic of China	cross-sectional	single	**C+: 120**	breast	MMSE **[intellectual functions: b117]**	Digit Span **[memory function: b144]**<br>VFT **[higher-level cognitive functions: b164]**<br>EPM **[memory functions: b144]**<br>TPM **[memory functions: b144]**	none	none
**37**	(Li et al., 2018)	**Asia** /People's Republic of China	cross-sectional	single	**C+: 28**<br>HC: 29	breast	MMSE **[intellectual functions: b117]**	Digit span **[attention functions: b140]**<br>VFT **[higher-level cognitive functions: b164]**	none	MRI-VBM

**38**	(Liu et al., 2018)	**Asia** /People's Republic of China	cross-sectional	single	**C+: 120**<br>HC: 120	breast	MMSE **[intellectual functions: b117]**	none	RMQ/PMQ	none
**39**	(Magnuson et al., 2019)	**North America /**United States	longitudinal	multi	**C+: 376**<br>HC: 234	breast	none	CANTAB **[intellectual functions: b117]**<br>COWA **[mental functions of language: b167]**<br>TMT **[attention functions: b140]**<br>HVLT-R **[memory functions: b144]**	FACT-Cog	none
**40**	(Miao et al., 2016)	**Asia** /People's Republic of China	cross-sectional	single	**C+: 23**<br>HC: 26	breast	MoCA **[intellectual functions: b117]**	Stroop **[higher-level cognitive functions: b164]**	none	rs-MRI
**41**	(Minisini et al., 2008)	**Europe** /Italy	longitudinal	single	**C+: 16**<br>C-: 45 (32/13)	breast, colorectal, and other	MMSE **[intellectual functions: b117]**	CAMCOG **[intellectual functions: b117]**	none	None
**42**	(Mo et al., 2017)	**Asia** /People's Republic of China	longitudinal	single	**C+: 19**<br>HC: 11	breast	MoCA **[intellectual functions: b117]**	Digit symbol (WAIS-III) **[psychomotor functions: b147]**<br>Digit span (WAIS-III) **[attention functions: b140]**<br>AVLT **[memory functions: b144]**	FACT-Cog	rs-fMRI<br>MRI-DTI
**43**	(Natori et al., 2015)	**Asia** /Japan	cross-sectional	single	**C+: 76**	breast	JART **[intellectual functions: b117]**	RCPM **[intellectual functions: b117]**	CFQ	none
**44**	(Ng et al., 2016)	**Asia** /Singapore	longitudinal	multi	**C+: 145**	breast	none	Headminder **[intellectual functions: b117]**	FACT-Cog	none
**45**	(Ng et al., 2018)	**Asia** /Singapore	longitudinal	multi	**C+: 145**	breast	none	Headminder **[intellectual functions: b117]**	FACT-Cog	none
**46**	(Nguyen et al., 2013)	**North America /**United States	cross-sectional	multi	**C+: 27**<br>C-: 30<br>HC: 30	breast	MMSE **[intellectual functions: b117]**	WAIS **[intellectual functions: b117]**<br>Reading subtest (WRAT) **[intellectual functions: b117]**<br>Digit span (WAIS-Ⅲ) **[attention functions: b140]**<br>Letter number sequencing (WAIS-Ⅲ) **[attention functions: b140]**<br>Arithmetic (WAIS-Ⅲ) **[attention functions: b140]**<br>TMT-A **[psychomotor functions: b147]**<br>COWA **[mental functions of language: b167]**<br>Boston naming test **[mental functions of language: b167]**<br>ROCF-copy **[perceptual functions: b156]**<br>Benton facial recognition test **[perceptual functions: b156]**<br>RAVLT **[memory functions: b144]**<br>ROCF-delay **[memory functions: b144]**<br>BVRT-R **[memory functions: b144]**<br>Sorting test **[higher-level cognitive functions: b164]**<br>TMT-B **[higher-level cognitive functions: b164]**	none	none
**47**	(Oh and Moon, 2019)	**Asia** /South Korea	cross-sectional	multi	C+: 67<br>HC: 66	stomach and colorectal	MMSE **[intellectual functions: b117]**	none	ECog	none

**48**	(Perrier et al., 2020)	**Europe** /France	longitudinal	single	<u>**C+:23**</u><br>HC: 27	breast	MMSE **[intellectual functions: b117]**	ESR **[memory functions: b144]**<br>BEM **[memory functions: b144]**<br>Digit span (WAIS-III) **[attention functions: b140]**<br>TMT-B **[higher-level cognitive functions: b164]**<br>VFT **[higher-level cognitive functions: b164]**<br>TMT-A **[psychomotor functions: b147]**	none	MRI-VBM
**49**	(Sales et al., 2019)	**South America /**Brazil	longitudinal	single	<u>**C+: 59**</u><br>C-: 26	colorectal	MMSE **[intellectual functions: b117]**	HVLT **[memory functions: b144]**<br>BVMT-R **[memory functions: b144]**<br>Digit span-forward **[attention functions: b140]**<br>TMT-A **[attention functions: b140]**<br>Digit symbol **[attention functions: b140]**<br>Digit span-backward **[higher-level cognitive functions: b164]**<br>TMT-B **[higher-level cognitive functions: b164]**<br>Stroop **[higher-level cognitive functions: b164]**<br>VFT (semantic) **[higher-level cognitive functions: b164]**<br>VFT (phonemic) **[higher-level cognitive functions: b164]**	ECog	MRI-DTI
**50**	(Schagen et al., 1999)	**Europe** /Netherlands	cross-sectional	multi	<u>**C+: 39**</u><br>C-: 34	breast	DART **[intellectual functions: b117]**	Digit span (WAIS) **[attention functions: b140]**<br>Digit symbol (WAIS) **[attention functions: b140]**<br>TMT-A **[attention functions: b140]**<br>D2 test **[attention functions: b140]**<br>Stroop **[higher-level cognitive functions: b164]**<br>TMT-B **[higher-level cognitive functions: b164]**<br>Fepsy visual reaction test **[psychomotor functions: b147]**<br>Fepsy binary choice test **[psychomotor functions: b147]**<br>Fepsy visual searching test **[psychomotor functions: b147]**<br>RAVLT **[memory functions: b144]**<br>ROCF-recall **[memory functions: b144]**<br>VFT **[mental functions of language: b167]**<br>ROCF-copy **[perceptual functions: b156]**<br>Fepsy finger-tapping **[mental functions, unspecified: b199]**	Cognitive Problems in Daily Life	none
**51**	(Schagen et al., 2008)	**Europe** /Netherlands	cross-sectional	single	<u>**C+: 70**</u><br>C-: 112 (57/55)	testicular	DART **[intellectual functions: b117]**	Digit span (WAIS) **[attention functions: b140]**<br>Digit symbol (WAIS) **[attention functions: b140]**<br>TMT-A **[attention functions: b140]**<br>D2 test **[attention functions: b140]**<br>Stroop **[higher-level cognitive functions: b164]**<br>TMT-B **[higher-level cognitive functions: b164]**<br>Fepsy visual reaction test **[psychomotor functions: b147]**<br>Fepsy binary choice test **[psychomotor functions: b147]**<br>Fepsy visual searching test **[psychomotor functions: b147]**<br>RAVLT **[memory functions: b144]**<br>ROCF-recall **[memory functions: b144]**<br>VFT **[mental functions of language: b167]**<br>ROCF-copy **[perceptual functions: b156]**<br>Fepsy finger-tapping **[mental functions, unspecified: b199]**	Cognitive Problems in Daily Life	none

**52**	(Scherwath et al., 2006)	**Europe** /Germany	cross-sectional	multi	**C+: 47** (24/23) HC: 29	breast	none	TMT-A **[psychomotor functions: b147]**<br>TMT-B **[higher-level cognitive functions: b164]**<br>TAP **[attention functions: b140]**<br>Go/No go **[attention functions: b140]**<br>D2 test **[attention functions: b140]**<br>Digit span (WMS-R) **[attention functions: b140]**<br>Spatial span (WMS-R) **[attention functions: b140]**<br>RAVLT **[memory functions: b144]**<br>ROCF **[memory functions: b144]**<br>RWT **[higher-level cognitive functions: b164]**<br>LPS **[higher-level cognitive functions: b164]**<br>HAWIE-R [**intellectual functions: b117]**	none	none
**53**	(Shen et al., 2019)	**Asia** /Taiwan	cross-sectional	multi	**C+: 19** HC: 20	breast	MMSE [**intellectual functions: b117]**	none	none	rs-fMRI
**54**	(Shilling et al., 2005)	**Europe** /United Kingdom	longitudinal	multi	**C+: 100** C-: 53 HC: 59	breast	NART [**intellectual functions: b117]**	Letter cancellation efficiency **[psychomotor functions: b147]**<br>AVLT **[memory functions: b144]**<br>Complex figure **[memory functions: b144]**<br>Logical memory (WMS) **[memory functions: b144]**<br>Letter-number sequencing (WMS) **[attention functions: b140]**<br>Digit span (WMS) **[attention functions: b140]**<br>Spatial span (WMS) **[attention functions: b140]**<br>Stroop **[higher-level cognitive functions: b164]**<br>NART [**intellectual functions: b117]**	none	none
**55**	(Silverman et al., 2007)	**North America /**United States	cross-sectional	single	**C: 16** (11/5) C-: 5 HC: 3	breast	NAART **[intellectual functions: b117]**	ROCF **[memory functions: b144]**	none	PET
**56**	(Simo et al., 2018)	**Europe** /Spain	cross-sectional	single	**C+: 17** C-: 15 HC: 18	lung	none	Vocabulary (WAIS-III) [**intellectual functions: b117]**<br>Boston naming test **[mental functions of language: b167]**<br>VFT **[higher-level cognitive functions: b164]**<br>TMT-A **[psychomotor functions: b147]**<br>TMT-B **[higher-level cognitive functions: b164]**<br>Digits (WAIS-III) **[attention functions: b140]**<br>ROCF copy **[perceptual functions: b156]**<br>ROCF delayed **[memory functions: b144]**<br>AVLT **[memory functions: b144]**	none	EEG
**57**	(Stouten-Kemperman et al., 2015)	**Europe** /Netherlands	cross-sectional	multi	**C+:43** (19/24) R+: 15 HC: 27	breast	NART [**intellectual functions: b117]**]	TMT-A **[attention functions: b140]**<br>TMT-B **[higher-level cognitive functions: b164]**<br>Digit symbol (WAIS-III) **[attention functions: b140]**<br>Stroop **[attention functions: b140]**<br>CVLT **[memory functions: b144]**<br>Visual Reproduction (WMS-R) **[memory functions: b144]**<br>WFT **[mental functions of language: b167]**<br>Fepsy finger tapping test **[mental functions, unspecified: b199]**	none	fMRI

**58**	(Tao et al., 2017)	**Asia** /People's Republic of China	cross-sectional	single	**C+: 33**<br>HC: 31	breast	MMSE [**intellectual functions: b117]**	Stroop **[higher-level cognitive functions: b164]**<br>TMT-A/B **[higher-level cognitive functions: b164]**	none	rs-MRI
**59**	(Van Dyk et al., 2021)	**North America /**United States	longitudinal	multi	**C+: 477**<br>HC: 435	breast	MMSE [**intellectual functions: b117]**<br><br>Reading subtest (WRAT-IV) [**intellectual functions: b117]**	TMT-A **[psychomotor functions: b147]**<br>TMT-B **[higher-level cognitive functions: b164]**<br>COWA **[higher-level cognitive functions: b164]**<br>Digit symbol **[psychomotor functions: b147]**<br>Logical memory **[memory functions: b144]**<br>NAB list A **[memory functions: b144]**	FACT-Cog	none
**60**	(Vardy et al., 2015)	**Multi** / Canada and Australia	longitudinal	multi	**C+:289**<br>(173/116)<br>C-: 73<br>HC: 72	colon/ rectum	none	Digit span **[attention functions: b140]**<br>Spatial span **[attention functions: b140]**<br>Letter number sequencing **[attention functions: b140]**<br>Digit symbol **[psychomotor functions: b147]**<br>TMT-A/B **[psychomotor functions: b147]**<br>HVLT-R **[memory functions: b144]**<br>BVMT-R **[memory functions: b144]**<br>CANTAB [**intellectual functions: b117]**	FACT-cog	none
**61**	(Wefel et al., 2014)	**North America /**United States	longitudinal	single	**C+: 50**<br>(25/25)<br>C-: 14)	testicular	none	Digit span **[attention functions: b140]**<br>Digit symbol **[psychomotor functions: b147]**<br>TMT-A **[psychomotor functions: b147]**<br>HVLT **[memory functions: b144]**<br>TMT-B **[higher-level cognitive functions: b164]**<br>COWA **[mental functions of language: b167]**<br>Grooved pegboard **[mental functions, unspecified: b199]**	none	none
**62**	(Williams et al., 2018)	**North America /**United States	cross-sectional	single	**C+: 22**	breast	none	CANTAB [**intellectual functions: b117]**	none	none

**63**	(Yamada et al., 2010)	**North America** /United States	cross-sectional	multi	**C+: 30** HC: 30	breast	Reading subtest (WRAT-III) [**intellectual functions: b117]** MMSE [**intellectual functions: b117]**	WAIS [**intellectual functions: b117]** Digit span (WAIS-III) **[attention functions: b140]** letter-number sequencing (WAIS-III) **[attention functions: b140]** COWA **[mental functions of language: b167]** TMT-A **[psychomotor functions: b147]** ROCF-copy **[perceptual functions: b156]** Boston naming test **[mental functions of language: b167]** Facial recognition test **[perceptual functions: b156]** RAVL **[memory functions: b144]** BVRT-R **[memory functions: b144]** Intradimensional/extradimensional shift task **[higher-level cognitive functions: b164]** ROCF-delay **[perceptual functions: b156]** TMT-B **[higher-level cognitive functions: b164]** Sorting test **[higher-level cognitive functions: b164]**	none	none
**64**	(Yao et al., 2017)	**North America** /Canada	longitudinal	single	**C+: 28**	breast	none	Stroop **[attention functions: b140]**	FACT-Cog PAOFI	none

Abbreviations: 15-WLT: 15-Word Learning Test , AFI: Attentional Function Index, AVLT: Auditory Verbal Learning test, BEM: Batterie d'efficience mnésique, BTA: Brief Test of Attention, BVMT-R: Brief Visuospatial Memory Test- Revised, BVRT-R: Benton Visual Retention Test-Revised, C+: Cancer group with chemotherapy group, C-: Cancer group without chemotherapy, CalCAP: California Computerized Assessment Package, CANTAB: Cambridge Neuropsychological Test Automated Battery, CCCs: Auditory Consonant Trigrams Test, CFQ: Cognitive Failures Questionnaire, CNS-VS: CNS Vital Signs, COWA: Controlled Oral Word Association Test, CPT: Continuous Performance Test, CVLT: California Verbal Learning Test, CWIT: Color-Word Interference Test, DART: Dutch Adult Reading test, D-KEFS: Delis-Kaplan Executive Function System, DOT: Design Organization Test, ECog: Everyday Cognition questionnaire, EEG: Electroencephalography, EPM: Event-based prospective memory task, ESR: Encoding Storage Retrieval, FACT-Cog: Functional Assessment of Cancer Therapy - Cognitive function, fMRI: functional Magnetic Resonance Imaging, HC: Healthy control group, HVLT-R: Hopkins Verbal Learning Test- Revised, JART: Japanese Adult Reading Test, LDST: Letter Digit Substitution Test, LPS: Leistungspruef system , MASQ: Multiple Ability Self-Report Questionnaire, MMSE: Mini Mental State Examination, MoCA: Montreal Cognitive Assessment, MRI-DTI: Magnetic Resonance Imaging-Diffusion Tensor Imaging, MRI-VBM: Magnetic Resonance Imaging-Voxel-Based Morphometry, MWT-B: Mehrfachwahl-Wortschatz-Intelligenz test, NAB: Neuropsychological Assessment Battery, NART: National Adult Reading Test, NAART: North American Adult Reading Test, NIH: National Institutes for Health, PASAT: Paced Auditory Serial Addition Test, PAOFI: Patient Assessment of Own Functioning Inventory, PET: Positron Emission Tomography, PPB: Purdue Pegboard test, PVT: Psychomotor Vigilance Test , RAVLT: Rey Auditory Verbal Learning Test, RBANS: Repeatable Battery of Adult Neuropsychological Status, RCPM: Raven's Colored Progressive Matrices, RMQ/PMQ: Retrospective memory and prospective memory questionnaires, ROCF: Rey-Osterrieth Complex Figure Test, rs-fMRI: resting state- functional Magnetic Resonance Imaging, RWT: Regensburg Word Fluency Test, SKT; Syndrom-Kurz test, TAP: Test Battery for Attentional Performance, TMT: Trail Making Test, TPM: Time-based prospective memory task, VFT: Verbal Fluency Test, WAIS: Wechsler Adult Intelligence Scale, WFT: Word Fluency Test, WMS-R: Wechsler Memory Scale- Revised, WRAT: Wide Range Achievement Test
